# FULL-MDS: Fluorescent Universal Lipid Labeling for Microfluidic Diffusional Sizing

**DOI:** 10.1021/acs.analchem.2c03168

**Published:** 2022-12-27

**Authors:** Jasmin Baron, Lena Bauernhofer, Sean R. A. Devenish, Sebastian Fiedler, Alison Ilsley, Sabrina Riedl, Dagmar Zweytick, David Glueck, Ariane Pessentheiner, Grégory Durand, Sandro Keller

**Affiliations:** † Biophysics, Institute of Molecular Biosciences (IMB), NAWI Graz, University of Graz, Humboldtstr. 50/III, Graz 8010, Austria; ‡ Field of Excellence BioHealth, University of Graz, Graz 8010, Austria; § BioTechMed-Graz, Graz 8010, Austria; ∥ The Paddocks Business Centre, Fluidic Analytics Ltd., Unit A, Cherry Hinton Road, Cambridge CB1 8DH, United Kingdom; ⊥ Equipe Synthèse et Systèmes Colloïdaux Bio-organiques, Unité Propre de Recherche et d’Innovation, Avignon Université, 301 rue Baruch de Spinoza, Avignon 84916 CEDEX 9, France; # CHEM2STAB, 301 rue Baruch de Spinoza, Avignon 84916 CEDEX 9, France

## Abstract

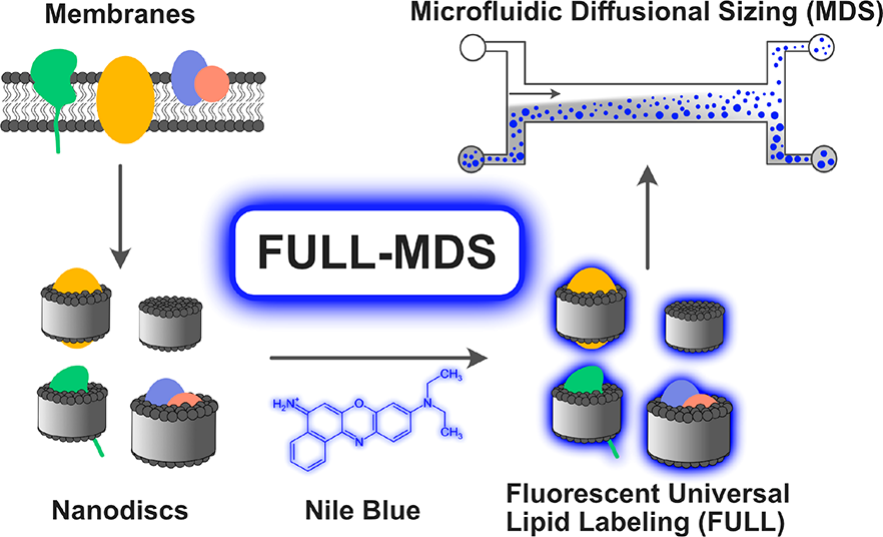

Microfluidic diffusional sizing (MDS) is a recent and powerful method for determining the hydrodynamic sizes and interactions of biomolecules and nanoparticles. A major benefit of MDS is that it can report the size of a fluorescently labeled target even in mixtures with complex, unpurified samples. However, a limitation of MDS is that the target itself has to be purified and covalently labeled with a fluorescent dye. Such covalent labeling is not suitable for crude extracts such as native nanodiscs directly obtained from cellular membranes. In this study, we introduce fluorescent universal lipid labeling for MDS (FULL-MDS) as a sparse, noncovalent labeling method for determining particle size. We first demonstrate that the inexpensive and well-characterized fluorophore, Nile blue, spontaneously partitions into lipid nanoparticles without disrupting their structure. We then highlight the key advantage of FULL-MDS by showing that it yields robust size information on lipid nanoparticles in crude cell extracts that are not amenable to other sizing methods. Furthermore, even for synthetic nanodiscs, FULL-MDS is faster, cheaper, and simpler than existing labeling schemes.

## Introduction

Microfluidic diffusional sizing (MDS) has recently been shown to reliably report hydrodynamic sizes of particles, such as lipid vesicles^1^ and nanoparticles like micelles or polymer-encapsulated nanodiscs.^2^ What is especially useful is that MDS can report the size of these particles even in complex mixtures such as crude cell extracts or biological fluids. This feature is particularly advantageous because the accuracy of other common methods, such as dynamic light scattering (DLS), is compromised by aggregates and sample heterogeneity, especially when determining the size of small particles.

Determining particle size with MDS requires that the particles of interest must be fluorescently labeled.^2−4^ To this end, fluorophores are covalently attached at the beginning of sample preparation.^5−7^ While this procedure is straightforward for purified proteins and synthetic lipid vesicles, it is not applicable to native nanoparticles obtained from cellular membranes. Therefore, it would be desirable to establish a simple fluorescent labeling method for MDS of native membrane nanoparticles that avoids covalent binding. A silicon-containing rhodamine dye has previously been used for the unspecific labeling and size determination of extracellular vesicles.^8^ However, this fluorescent dye is not available commercially, requires multistep synthesis, and needs to be activated by UV light for covalent attachment to lipid molecules.^8^ We report here a facile, sparse, and noncovalent labeling method for MDS whereby the readily available fluorophore, Nile blue, spontaneously partitions into the hydrophobic core of lipid nanoparticles. We show that the structure of these nanoparticles is maintained, for both synthetic nanoparticles and native nanoparticles derived from cellular membranes. We also show that the method is suitable even for crude cell extracts, which means that no special sample preparation or purification steps are required. Because the method should be suitable for all lipid-based nanoparticles, we have termed the method fluorescent universal lipid labeling for microfluidic diffusional sizing, FULL-MDS.

## Experimental Section

### Materials

All chemicals were obtained in the highest purity available. Glyco-DIBMA and *n*-dodecyl-β-d-maltoside (β-DDM) were kind gifts from Glycon Biochemicals (Luckenwalde, Germany). *N*-(2-Methyl-1,3-bis(*O*-β-d-glucose)propan-2-yl)-3-(dodecylthio)propanamide (DDDG) was synthesized as described elsewhere.^9^ 1,2-Dimyristoyl-*sn*-glycero-3-phosphocholine (DMPC) and 1-palmitoyl-2-oleoyl-*sn*-glycero-3-phosphocholine (POPC) were obtained from Lipoid (Ludwigshafen, Germany). The human melanoma cell line WM164 from metastatic lesions was kindly provided by Dr. Meenhard Herlyn (The Wistar Institute, Philadelphia, US). Dimethyl sulfoxide (DMSO) was purchased from VWR International (Vienna, Austria). Ethylenediaminetetraacetic acid (EDTA), Nile blue A ((Nile blue)_2_ sulfate), Na_2_CO_3_, NaCl, and tris(hydroxylmethyl)aminomethane (Tris) were obtained from Carl Roth (Karlsruhe, Germany). EDTA-free c0mplete protease inhibitor was obtained from Merck (Vienna, Austria). RPMI 1640 medium (including GlutaMAX), fetal bovine serum (FBS), Accutase, and DPBS buffer (Dulbecco’s phosphate-buffered saline) were obtained from Thermo Fisher Scientific (Gibco, Waltham, US). All solutions were prepared with ultrapure water (18 MΩ cm). Experiments with synthetic nanodiscs were performed in buffer containing 50 mM Tris and 200 mM NaCl at pH 7.4. Experiments with native nanodiscs were performed in buffer containing 50 mM Tris and 150 mM NaCl at pH 7.4.

### Stock Solutions

A stock solution of Glyco-DIBMA was prepared by dissolving Glyco-DIBMA powder in water and sonicating for 10 min at 50 °C. The resulting suspension (4 mL) was then transferred into a 5 mL QuixSep microdialysis capsule (Carl Roth) using a Spectra/Pore 3 dialysis membrane with a molar-mass cutoff of 3.5 kg/mol (Spectrum Laboratories, Rancho Dominguez, US). Dialysis was carried out for 20 h at room temperature under slow circling, with buffer exchange after 10 h. The polymer stock was then filtered through a polyvinylidene fluoride syringe filter (0.22 μm, Carl Roth). Finally, the Glyco-DIBMA concentration was determined by measuring the refractive index of the stock solution with an Abbemat 500 refractometer (Anton Paar, Graz, Austria). A stock solution of the amphiphile DDDG was obtained by suspending a defined amount of DDDG in buffer. The stock solution was filtered through a polyvinylidene fluoride syringe filter (0.22 μm, Carl Roth). A stock solution of the detergent β-DDM was obtained by dissolving a defined amount of β-DDM in buffer. A Nile blue stock solution was prepared by dissolving (Nile blue)_2_ sulfate in 100% DMSO to a final Nile blue dye concentration of 20 μM (corresponding to a (Nile blue)_2_ sulfate concentration of 10 μM).

### Preparation of Synthetic Nanodiscs

Large unilamellar vesicles were prepared by dissolving synthetic phospholipids (DMPC or POPC) in buffer and shaking for 30 min at 35 °C and 800 rpm. The POPC suspension was extruded with a LiposoFast extruder (Avestin, Mannheim, Germany) at room temperature, while for DMPC suspensions a Mini-Extruder (Avanti Polar Lipids, Alabaster, US) at 35 °C was used to keep this saturated lipid above its phase-transition temperature. Extrusion was performed with at least 13 repeats through two stacked polycarbonate membranes with a pore diameter of 100 nm (Cytiva, Freiburg, Germany). Synthetic nanodiscs or micelles were prepared at a final lipid concentration of 1 mM and different solubilizing agent/lipid mass ratios (*m*/*m*), where the solubilizing agents were the amphiphilic polymer Glyco-DIBMA, the nanodisc-forming amphiphile DDDG, or the conventional, micelle-forming detergent β-DDM.

### Nile Blue Emission Spectra

Emission spectra of 10 μM Nile blue in buffer or synthetic nanodiscs were measured on a Cary Eclipse fluorescence spectrophotometer (Agilent Technologies, Vienna, Austria) using a SUPRASIL quartz cuvette (Hellma, Müllheim, Germany) with a cross-section of 10 mm × 10 mm. Samples were excited at 620 nm, and emission spectra were recorded between 620 and 750 nm with excitation and emission slit widths of 5 nm at a scan rate of 10 nm s^–1^. For each sample, three emission spectra were recorded and averaged.

### Solubilization of *Escherichia coli* Membranes

*E. coli* cells (2 g) were resuspended in an ice-cold aqueous solution containing 100 mM Na_2_CO_3_ (20 mL) and ultrasonicated twice for 10 min with a 10 min break in between. All sample preparation was carried out on ice. Cell debris and unbroken cells were removed by centrifuging at 3000*g* for 30 min at 4 °C. The supernatant was then ultracentrifuged at 100 000*g* for 1 h at 4 °C using a TLA100.3 rotor (Beckman Coulter, Vienna, Austria). The resulting cell-membrane pellets were washed three times with Tris buffer (50 mM Tris, 200 mM NaCl, 2 mM EDTA, pH 7.4), resuspended in the same buffer, and treated with Glyco-DIBMA to obtain a final Glyco-DIBMA concentration of 1% (*w*/*v*). These samples were shaken at 500 rpm overnight at room temperature before being centrifuged at 25 000*g* for 20 min at 4 °C. The nanodisc-containing supernatant was then analyzed with MDS and DLS.

### Solubilization of Human Membranes

For experiments with eukaryotic membranes, we used human melanoma cell line WM164 from metastatic lesions. The cell line was cultured in RPMI 1640 medium with GlutaMAX (Gibco, Thermo Fisher Scientific, US) supplemented with 2% (*v*/*v*) fetal bovine serum (FBS) under an atmosphere of 5% (*v*/*v*) CO_2_ at 37 °C. At 90% confluence, cells were passaged with Accutase (Gibco, Thermo Fisher Scientific). For solubilization, cells were grown in cell culture flasks with a surface area of 75 cm^2^ (Thermo Scientific Nunc EasYFlas, Thermo Fisher Scientific). At 90% confluence, the medium was removed, and cells were thoroughly washed twice with DPBS buffer without CaCl_2_ and MgCl_2_ (Gibco) and once with sterile-filtered sample buffer containing 150 mM NaCl and 50 mM Tris at pH 7.4. Cells were then solubilized within the culture flasks using Glyco-DIBMA or DDDG (0.1% (*w*/*v*)) in sample buffer supplemented with 0.5 × c0mplete protease inhibitor for 90 min at room temperature, under gentle agitation. Samples were then centrifuged at 25 000*g* for 20 min at 4 °C. The resulting supernatants containing native nanodiscs were then analyzed with MDS and DLS.

### Fluorescent Labeling of Synthetic and Native Nanodiscs for MDS

Synthetic nanodisc samples were labeled by adding Nile blue stock solution to give a final dye concentration of 25–50 nM for nanodiscs prepared from either DDDG or Glyco-DIBMA. Samples were then incubated for 1 h at 25 °C for POPC- and 35 °C for DMPC-containing nanodiscs. The β-DDM stock solution was diluted to 0.5 mM and labeled by adding Nile blue stock solution to give a final dye concentration of 50 nM, followed by incubation for 1 h at room temperature. Native nanodisc samples containing 5 mg/mL *E. coli* membrane were labeled by adding Nile blue stock solution to give a final dye concentration of 100 nM. Native nanodisc samples containing WM164 membrane were labeled by adding Nile blue stock solution to give a final dye concentration of 250–500 nM. Both types of native nanodisc samples were then incubated for 1 h at room temperature.

### Microfluidic Diffusional Sizing (MDS)

MDS measurements were performed at room temperature on a Fluidity One-W Serum (Fluidic Analytics, Cambridge, UK) using a 647 nm LED with the size range set at 2–20 nm. The sample (5 μL) was loaded onto the chip before the chip was inserted into the MDS device. A schematic of the measurement principle is included in Figure S1. Briefly, the MDS device accommodates a microfluidic chip with two channels. One channel contains the fluorescently labeled sample to be analyzed, while the other channel contains an auxiliary fluid (i.e., water). The channels are initially separate but later merge, so particles are free to diffuse between the two coflowing laminar fluid streams. Smaller particles diffuse faster than larger ones, so smaller particles will become more evenly distributed between the two fluid streams within a given flow time. After a defined contact time, the two fluid streams are separated again in two outlet channels, and the concentration of fluorescent analyte particles in each channel is measured. In the following, particles detected in the original analyte channel will be referred to simply as “undiffused particles”, while particles detected in the other channel will be referred to as “diffused particles”. The hydrodynamic size of the analyte particles is then obtained from the ratio of diffused to undiffused particles, that is, from the ratio of fluorescence signals measured in the two channels. Assuming that the degree of fluorescence labeling is proportional to the mass of the diffusing particles, MDS thus provides the mass-average hydrodynamic size of the labeled species in the sample.

### Dynamic Light Scattering (DLS)

Size distribution measurements by DLS were performed on a Nano Zetasizer ZS90 (Malvern Instruments, Malvern, UK) equipped with a 633 nm He–Ne laser and a detection angle of 90° using 45 μL quartz glass cuvettes with a cross-section of 3 mm × 3 mm (Hellma, Müllheim, Germany). Measurements were performed at 25 °C for POPC, 35 °C for DMPC, and 37 °C for native nanodiscs obtained from crude extracts. Each sample was measured with the software-optimized attenuator position including 12 runs of 10 s per run.

### Size-Exclusion Chromatography (SEC)

SEC was performed on a fast protein liquid-chromatography (FPLC) instrument (Shimadzu, Japan) equipped with a degasser (DGIU20A), a pump (LC20AI), a UV/vis detector (SPDM40), a fluorescence detector (RF20A), and a fraction collector (FRC10A9). Samples in aqueous buffer (50 mM Tris, 150 mM NaCl, pH 7.4) were loaded via an injector (Rheodyne 9725i, IDEX, US) equipped with a 250 μL loop and eluted through a Superose 6 Increase 10/300 GL column (Cytiva, Austria) at a flow rate of 0.3 mL/min. For SEC, final concentrations of 4 mM lipid for synthetic nanodiscs and 50 mg/mL bio wet mass for native nanodiscs were used. The final Nile blue concentration was 10 μM. The absorbance of Nile blue was detected at 630 nm, and fluorescence was measured at 680 nm upon excitation at 640 nm.

## Results and Discussion

### Nile Blue as a Fluorophore for FULL-MDS

**Figure 1:**
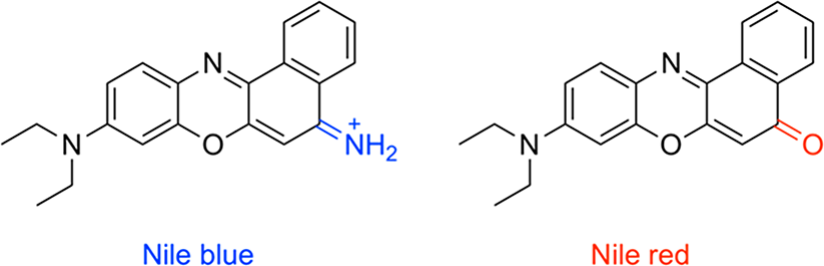
Chemical structures of fluorescent dyes. The structure of Nile blue (left) is similar to that of the commonly used lipid-staining dye Nile red (right) and was thus selected for FULL-MDS.

We selected Nile blue (Figure 1) as fluorophore for FULL-MDS because it is lipophilic, readily available, and intensely fluorescent in nonpolar environments but dark in polar environments (Figure S2).^10,11^ It has a broad absorption spectrum (490–680 nm)^10,12,13^ and can be excited at 647 nm, the excitation wavelength of the latest-generation MDS devices. Furthermore, Nile blue is similar in structure and properties to Nile red (Figure 1), which is known to be an excellent lipid stain.^14−17^ Thus, we reasoned that Nile blue should have similar lipid-staining abilities.

### Suitability of Nile Blue

**Figure 2:**
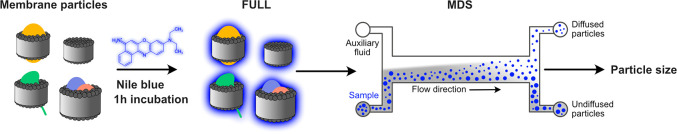
General workflow of FULL-MDS. First, lipid nanoparticles (e.g., nanodiscs, micelles) are prepared by mixing lipid-containing material (e.g., vesicles, cells, or whole tissue) with a solubilizing agent (e.g., nanodisc-forming polymer or small-molecule amphiphile or micelle-forming detergent). Nile blue is added to this sample, which is then incubated for 1 h. Finally, the labeled sample is measured by MDS. This labeling scheme works for nanodiscs (as shown in the main text) as well as for other lipid-containing nanoparticles such as micelles (Figure S3).

We first investigated if Nile blue would be suitable for sparse, noncovalent fluorescent labeling of lipid-containing nanodiscs. To this end, we examined its ability to partition into the hydrophobic core of the lipid environment. We prepared POPC-containing nanodiscs encapsulated by Glyco-DIBMA. Nile blue was simply added after nanodisc formation and was allowed to partition into the nanodiscs for 1 h before MDS measurements were made. The schematic workflow is shown in Figure 2.

**Figure 3:**
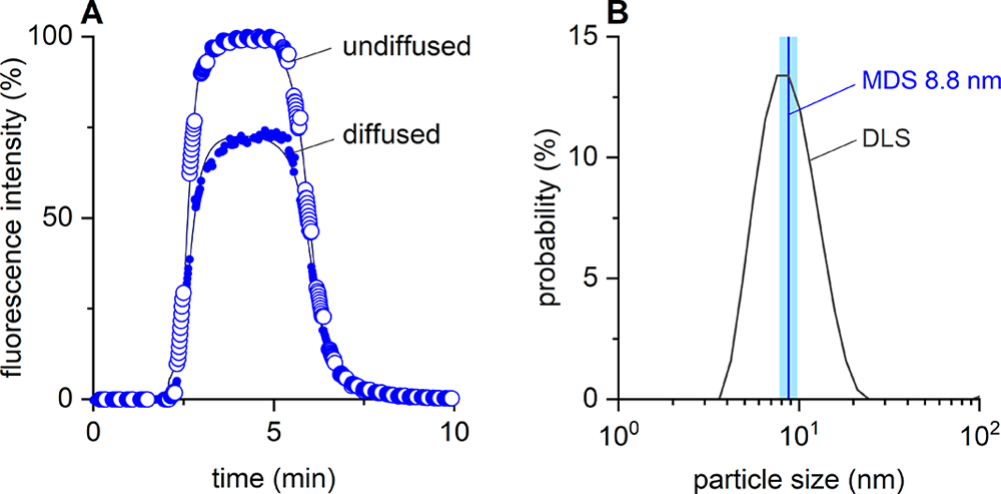
MDS and DLS of POPC-containing nanodiscs encapsulated by Glyco-DIBMA at a Glyco-DIBMA/POPC mass ratio of 2. (A) MDS traces of nanodiscs containing 1 mM POPC labeled with 25 nM Nile blue. Shown are experimental traces for diffused (●) and undiffused particles (○) as well as corresponding fits (lines). The observation of fluorescence confirms that the fluorophore molecules are integrated within the nanodiscs and are thus suitable for sparse, noncovalent labeling. (B) Comparison of hydrodynamic sizes determined by either MDS or DLS shows good agreement. The blue vertical line indicates the size measured by MDS, while the light-blue shaded band encompasses ±1 standard deviation determined from three MDS measurements. The black curve represents the intensity-weighted particle-size distribution derived from DLS.

MDS traces of POPC/Glyco-DIBMA nanodiscs labeled with Nile blue are shown in Figure 3. The observation of an intense fluorescence signal confirmed that Nile blue is integrated into the nanodiscs, such that the hydrodynamic sizes of the nanodiscs can be determined by MDS. In MDS, the ratio of the fluorescence intensities measured in traces of diffused versus undiffused particles provides the particle size, which was 8.8 nm for our POPC/Glyco-DIBMA nanodiscs labeled with Nile blue (Figure 3A). As a negative control, Nile blue in aqueous buffer without lipid-containing nanodiscs did not exhibit significant fluorescence (Figure S2) and, consequently, could not be sized by MDS. Once Nile blue is incorporated into lipid-bilayer nanodiscs, however, its fluorescence signal could be reliably detected and used for size determination even at low nanomolar dye concentrations (Figure S4). Moreover, the fluorescence emission intensity of Nile blue increased linearly with its concentration over at least 2 orders of magnitude (Figure S4). We also analyzed the nanodisc sample by using DLS (Figure 3B). A direct comparison of the size determined by MDS and the size distributions derived from DLS (Figure 3B) indicates that the hydrodynamic sizes reported by both methods agree well with one another. In conclusion, these results show that Nile blue is highly suitable as a sparse, noncovalent fluorescent label for FULL-MDS (Figure S5) and that MDS can be used as an alternative method to DLS for determining nanoparticle size.

### FULL-MDS Can Reveal Small Differences in Particle Size

**Figure 4:**
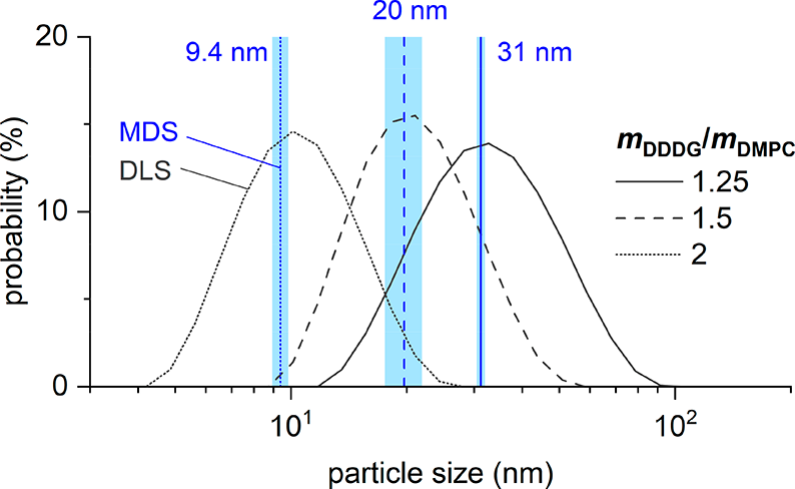
MDS and DLS of DMPC-containing nanodiscs encapsulated by DDDG at different DDDG/DMPC mass ratios. Comparison of MDS and DLS measurements of nanodiscs containing 1 mM DMPC at DDDG/DMPC mass ratios of 1.25, 1.5, and 2.0 labeled with 25–50 nM Nile blue. The blue vertical lines indicate the sizes measured by MDS, and each of the light-blue shaded bands encompasses ±1 standard deviation determined from three MDS measurements. Black curves represent intensity-weighted particle-size distributions derived from DLS. Hydrodynamic sizes determined by MDS and DLS are in good agreement.

To investigate whether FULL-MDS can reliably detect small changes in particle size, we prepared DMPC-containing nanodiscs encapsulated by the small-molecule amphiphile DDDG because nanodisc size can be tightly controlled by varying the DDDG/DMPC ratio.^7^ Thus, nanodiscs were generated at DDDG/DMPC mass ratios of 1.25, 1.5, and 2.0. Again, labeling with Nile blue was carried out after nanodisc formation. Indeed, MDS experiments revealed hydrodynamic nanodisc sizes of 31.2, 19.7, and 9.4 nm (Figure 4) at these three mass ratios, respectively. DLS measurements on the same samples agreed with the MDS measurements, with particle-size distributions peaking at 31.9, 20.6, and 10.3 nm.

To further test the robustness of FULL-MDS with different nanodisc-forming amphiphiles, lipids, and amphiphile/lipid ratios, we prepared more nanodiscs from either DMPC or POPC using either Glyco-DIBMA or DDDG at different ratios (Figure S6). In all cases, the nanodiscs were labeled with Nile blue after sample preparation. MDS results revealed particle sizes within the ranges expected and agreed well with DLS results, regardless of the lipid, amphiphile, and mass ratio used (Figures S6 and S7). These results show that Nile blue labeling is independent of lipid type and solubilizing agent, enabling MDS to reveal small differences in particle size for a range of particle systems.

### Measuring Nanoparticles in Crude Cell Extracts

**Figure 5:**
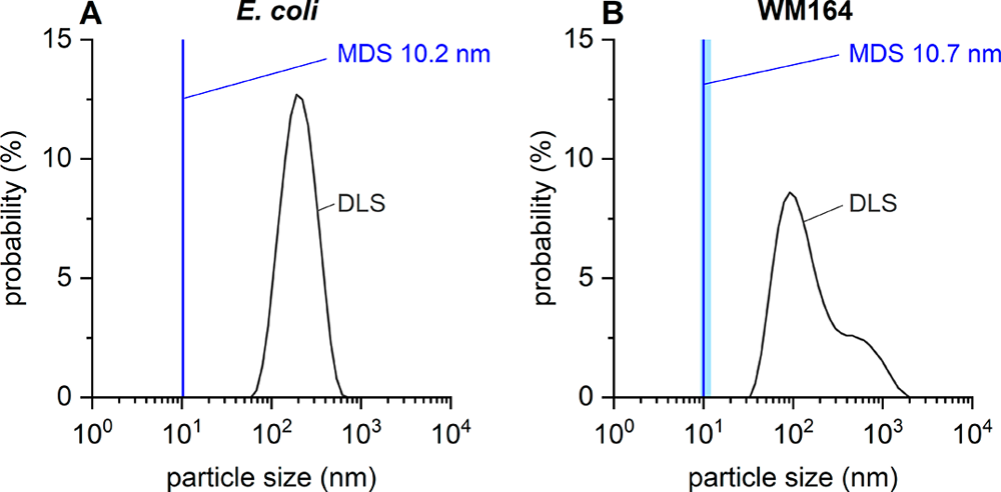
MDS and DLS of native nanodiscs prepared from either (A) *E. coli* membranes or (B) human melanoma (WM164) cells using Glyco-DIBMA as a solubilizing agent. Native nanodiscs derived from *E. coli* membranes were labeled with 100 nM Nile blue, and native nanodiscs from WM164 membranes were labeled with 25–50 nM Nile blue. The figure shows that FULL-MDS, but not DLS, is suitable for measuring lipid nanoparticles in complex mixtures because MDS is not affected by the heterogeneous nature of crude cell extracts. The blue vertical lines indicate the sizes measured by MDS, and each of the light-blue shaded bands encompasses ±1 standard deviation determined from three MDS measurements; note that, for native nanodiscs obtained from *E. coli* membranes, this light-blue band is barely visible. Black curves represent intensity-weighted particle-size distributions derived from DLS.

Having established that FULL-MDS can reliably provide accurate and precise measurements across a range of lipid types and sizes, we wondered whether FULL-MDS could be used to measure nanoparticle sizes in complex mixtures such as crude cell extracts. On the one hand, a key advantage of MDS is its ability to neglect large particles and robustly measure the size of nanoparticles. On the other hand, crude cell extracts have not previously been analyzable by standard MDS, because this would have required covalent fluorescent labeling. Furthermore, DLS is also unsuitable for analyzing crude cell extracts because the large particles disproportionately dominate the scattering intensity and skew the size measurements. Thus, to investigate whether FULL-MDS is feasible for detecting and sizing nanoparticles in crude cell extracts, we solubilized *E. coli* membranes with Glyco-DIBMA as reported previously.^18^ The resulting native nanodiscs were labeled with Nile blue and were measured by MDS to be 10.2 nm in size (Figure 5A and Table S1), which is similar to previous results.^18^ In stark contrast, DLS measurements on the same sample were disproportionately dominated by large particles, measurements returning an apparent size of >100 nm (Figure 5A and Table S1) and showing no indication of the nanodiscs detected by MDS and known to be present in these samples from other methods, including electron microscopy (EM).^18^

We next turned to eukaryotic cells in the form of a human melanoma cell line (WM164). WM164 human melanoma cells were solubilized with 0.1% Glyco-DIBMA or DDDG, and the resulting native nanodiscs were labeled with Nile blue and measured by MDS and DLS. Here too, we expected a hydrodynamic size of around 10 nm for the native nanodiscs. Indeed, MDS measurements afforded a particle size of 10.7 nm, whereas DLS failed to detect these nanodiscs, instead returning a broad, multimodal size distribution dominated by the much larger aggregates that were present due to incomplete solubilization (Figure 5B and Table S1).

Thus, we conclude that FULL-MDS can reliably measure the hydrodynamic size of lipid nanoparticles in crude, heterogeneous cell extracts of both prokaryotic and eukaryotic origin (Figure 5) and that these measurements are not impeded by the presence of much larger particles. In contrast, such results could not be achieved with DLS because the larger particles dominate the light-scattering intensity even though those larger particles account for only a small fraction of the lipid mass in the sample.

### Validation of Results by Size-Exclusion Chromatography

**Figure 6:**
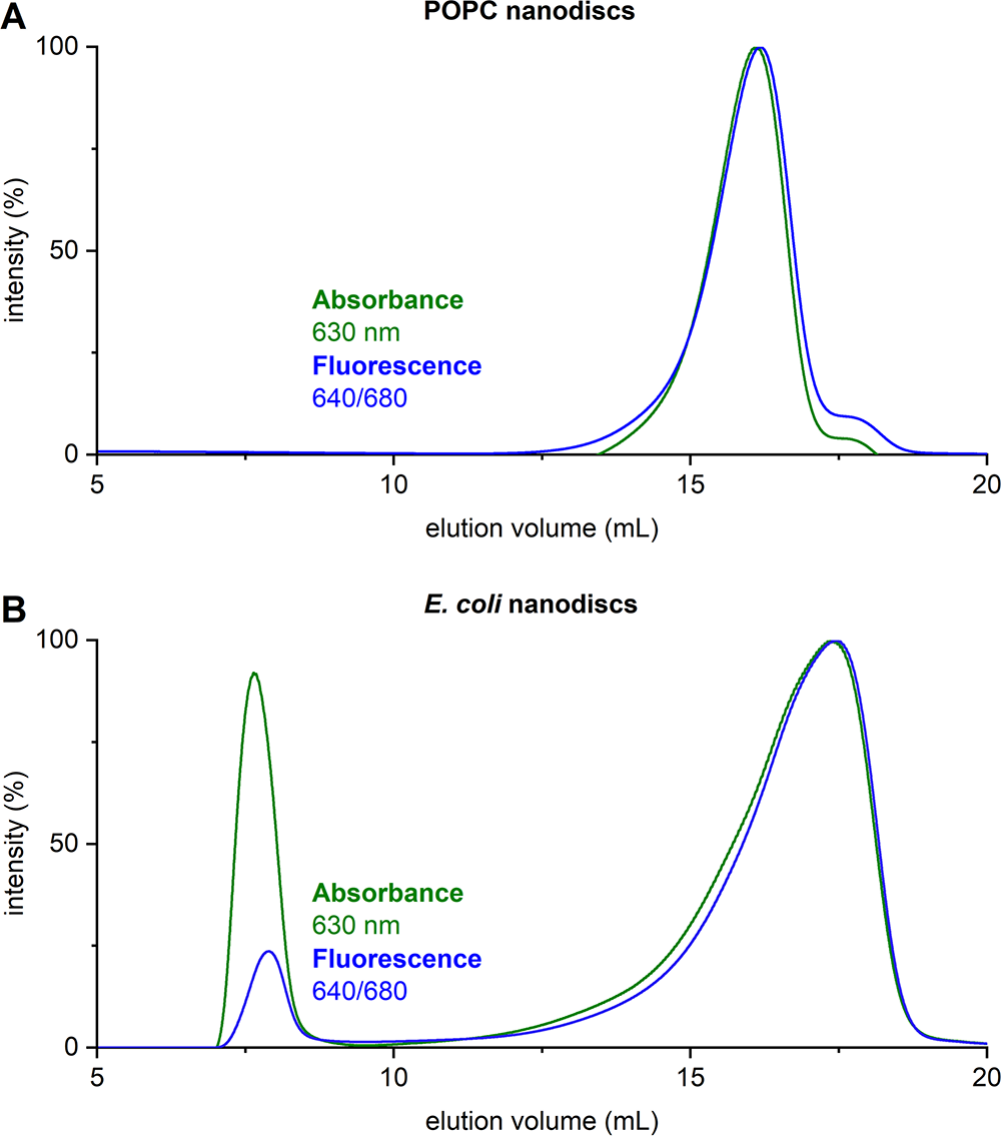
SEC of nanodiscs prepared from either (A) POPC vesicles or (B) *E. coli* membranes using Glyco-DIBMA as a solubilizing agent. (A) Nile blue absorption (green) and emission (blue) profiles in POPC-containing synthetic nanodiscs reveal a single peak at an elution volume of 16 mL, confirming that only one population of nanosized particles is present. (B) Nile blue absorption (green) and emission (blue) profiles in native nanodiscs containing *E. coli* membrane fragments reveal two peaks (elution volumes of 6–8 and 15–18 mL), indicating that, along with the nanosized particles, larger lipid-containing particles are also present.

To further validate the MDS results, we analyzed selected samples by size-exclusion chromatography (SEC). SEC traces for POPC-containing nanodiscs encapsulated by Glyco-DIBMA were obtained by recording both the absorbance at 630 nm and the emission at 680 nm of Nile blue (Figure 6A). The single sharp peak in each SEC trace confirms the presence of a single, homogeneous population of nanosized particles. After SEC, samples at an elution volume of 15–18 mL were measured with MDS again, revealing an average particle size of (9.1 ± 0.1) nm, consistent with the previous size measurements (Figure 3).

We also analyzed the native nanodiscs made from *E. coli* crude cell extracts by SEC, again by detecting both the absorbance and the emission of Nile blue (Figure 6B). Both traces revealed two sharp peaks. We subjected samples eluted at 15–18 mL to MDS analysis, with the measured particle size (8.2 ± 0.1) nm again confirming the presence of nanodiscs, as shown in the previous MDS analyses (Figure 5). The peak across an elution volume of 6–8 mL indicates the presence of much larger lipid-containing particles that were not completely solubilized by Glyco-DIBMA or removed by centrifugation. This peak was detected because Nile blue labeling is not specific to nanodiscs but is universally applicable to lipid-containing particles (Figure S3). Previous SEC studies have revealed similar results, with aggregate peaks at similar elution volumes detected by monitoring the extinction at 280 nm.^18,19^ Taken together, these SEC results support our assertion that the DLS measurements on the same samples (Figure 5) were unable to detect nanodiscs because they were overshadowed by aggregates present due to incomplete solubilization. By contrast, and more importantly, the SEC results highlight the usefulness of our FULL-MDS method, which can selectively measure nanosized lipid-based particles, even in the presence of much larger particles in crude cell extracts.

## Conclusions and Outlook

In this study, we developed fluorescent universal lipid labeling for microfluidic diffusional sizing (FULL-MDS). The key advantages of our sparse, noncovalent labeling method are that lipid nanoparticles in complex solutions or suspensions can be analyzed by MDS with very little purification and without tedious and invasive covalent labeling. Consequently, FULL-MDS can be used to determine nanoparticle sizes not only in well-defined systems such as synthetic lipid-bilayer nanodiscs but also in complex membrane systems such as native nanodiscs obtained from crude cell extracts. This becomes especially important in downstream applications such as optical spectroscopy and microfluidics, which can handle crude extracts and other complex samples provided that the particle size is in the nanometer range. For the specific case of self-assembled nanodiscs formed directly from cellular membranes, FULL-MDS enables the reliable detection of nanodiscs even in the presence of unsolubilized aggregates, which are commonly encountered in crude cell extracts. Furthermore, MDS requires only small sample volumes and low sample and fluorophore concentrations, making this method inexpensive. As sample preparation is rapid and simple, FULL-MDS is suitable even for nonspecialist laboratories interested in applications as diverse as studying extracellular vesicles or native membrane-protein libraries derived directly from cellular membranes.

## Supporting Information Available

Working principle of MDS, Nile blue emission spectra, additional results on micelles, linearity test of MDS signal, correlation of MDS and DLS results, and additional results on synthetic and native nanodiscs (PDF)
