# Supplementary material for: FULL-MDS: Fluorescent Universal Lipid Labeling for Microfluidic Diffusional Sizing
Source: Anal Chem. 2022 Dec 27;95(2):587–93. doi: 10.1021/acs.analchem.2c03168 (PMC9850350; doi:10.1021/acs.analchem.2c03168)
Supplement: Supplementary file 1 — ac2c03168_si_001.pdf [file ac2c03168_si_001.pdf]

Supporting Information

FULL-MDS: Fluorescent Universal Lipid Labeling for Microfluidic Diffusional Sizing

Jasmin Baron,[a,b,c] Lena Bauernhofer,[a,b,c] Sean R.A. Devenish,[d] Sebastian Fiedler,[d] Alison Ilsley,[d] Sabrina Riedl,[a,b,c] Dagmar Zweytick,[a,b,c] David Glueck,[a,b,c] Ariane Pessentheiner,[a,b,c] Grégory Durand,[e,f] and Sandro Keller[a,b,c,*]

[a] Biophysics, Institute of Molecular Biosciences (IMB), NAWI Graz, University of Graz, Humboldtstr. 50/III, 8010 Graz, Austria

[b] Field of Excellence BioHealth, University of Graz, 8010 Graz, Austria

[c] BioTechMed-Graz, 8010 Graz, Austria

[d] Fluidic Analytics Ltd, Unit A, The Paddocks Business Centre, Cherry Hinton Road, Cambridge CB1 8DH, United Kingdom

[e] Equipe Synthèse et Systèmes Colloïdaux Bio-organiques, Unité Propre de Recherche et d'Innovation, Avignon Université, 301 rue Baruch de Spinoza, 84916 Avignon cedex 9, France

[f] CHEM2STAB, 301 rue Baruch de Spinoza, 84916 Avignon cedex 9, France

* Corresponding author, e-mail address: sandro.keller@uni-graz.at

Table of Contents

**Figure S1:**	Working principle of MDS	**2**
**Figure S2:**	Nile blue emission spectra	**2**
**Figure S3:**	MDS and DLS of β-DDM micelles	**3**
**Figure S4:**	Linearity of fluorescence intensity measured by MDS	**3**
**Figure S5:**	MDS and DLS of POPC nanodiscs solubilized with Glyco-DIBMA	**4**
**Figure S6:**	MDS and DLS of POPC and DMPC nanodiscs	**5**
**Figure S7:**	Correlation of particle sizes measured by MDS and DLS	**6**
**Table S1:**	MDS and DLS results for native nanodiscs made from cellular membranes	**6**

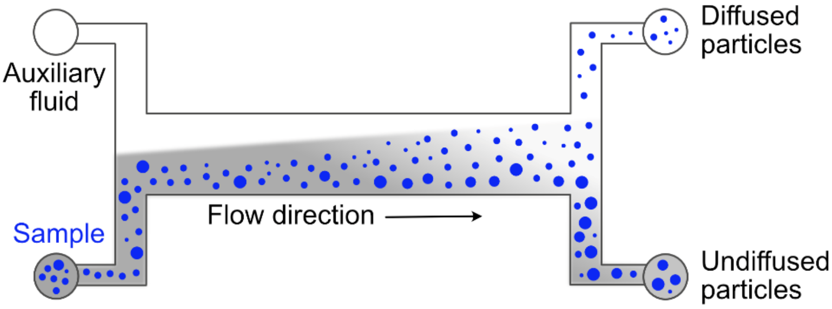

Figure S1: Working principle of MDS. The microfluidic chip has two channels. One contains the fluorescently labeled sample to be analyzed, while the other contains an auxiliary fluid (i.e., water). The channels are initially separate but later merge, so particles are free to diffuse between the two channels. Smaller particles diffuse faster than larger ones, so smaller particles will become more distributed between the two channels within a given flow time. After the separation of the two channels, the hydrodynamic size of the diffusing particles is obtained from the ratio of fluorescence signals measured in the two channels.

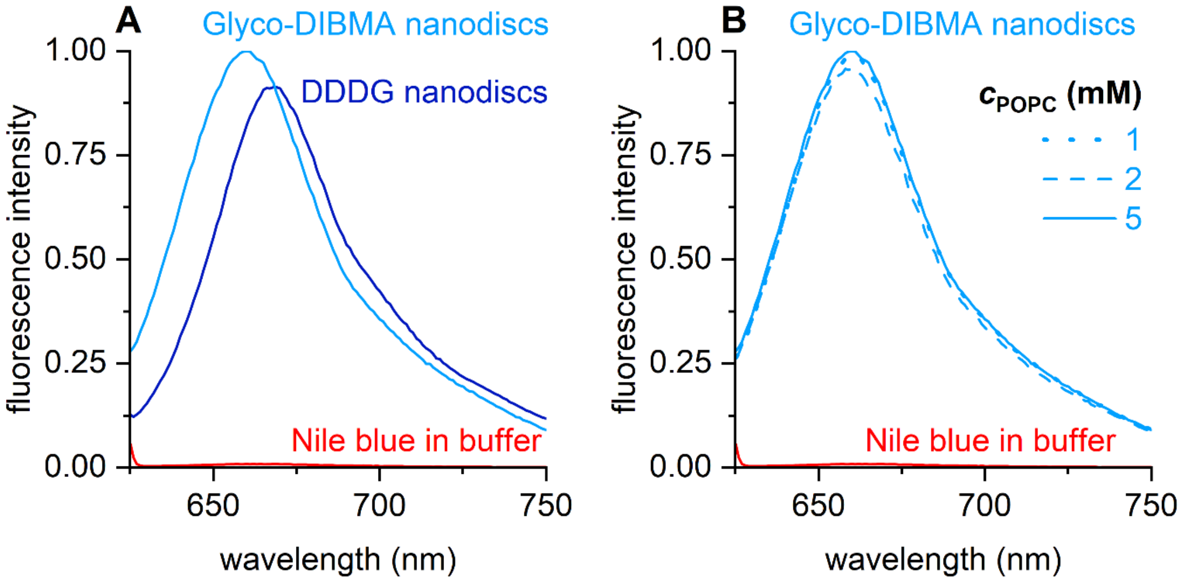

Figure S2: Nile blue emission spectra. (A) 10 µM Nile blue in buffer or in 5 mM POPC nanodiscs encapsulated by Glyco-DIBMA (polymer/lipid mass ratio 2.0) or DDDG (amphiphile/lipid mass ratio 2.5). Nile blue is intensely fluorescent in nanodiscs but does not fluoresce in buffer. **(B)** 10 µM Nile blue in the presence of various concentrations of POPC/Glyco-DIBMA nanodiscs (mass ratio 2.0), specified in terms of lipid concentrations. The fluorescence intensity of Nile blue is independent of the lipid concentration.

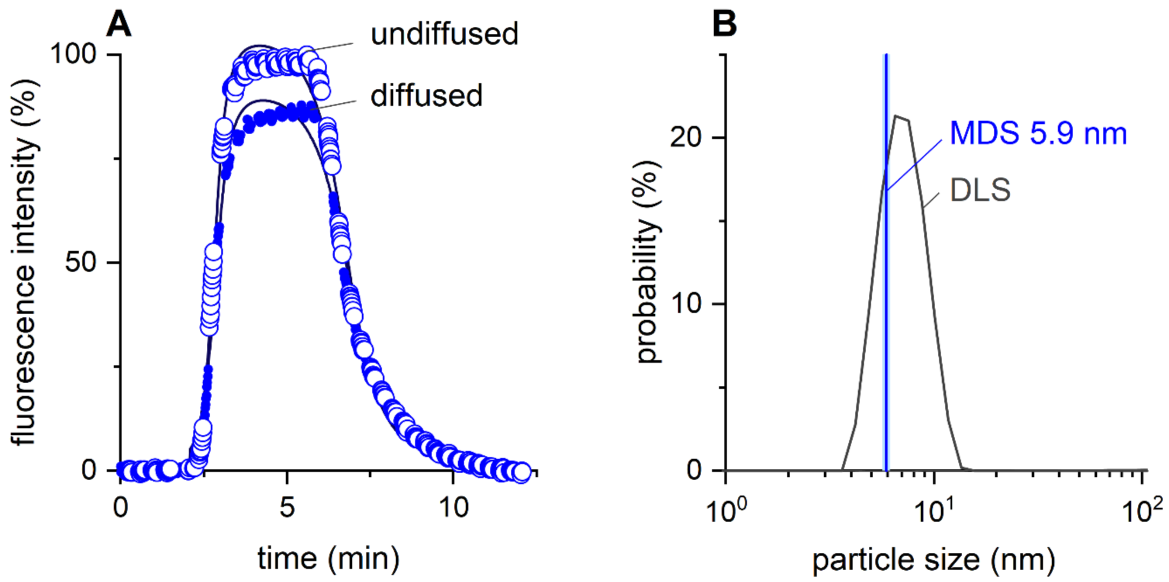

Figure S3: MDS and DLS of β-DDM micelles. (**A**) 0.5 mM β-DDM micelles labeled with 50 nM Nile blue (*n* = 3). (**B**) Comparison of MDS and DLS measurements. The blue vertical line indicates the size measured by MDS, while the light-blue shaded band encompasses ±1 standard deviation determined from three MDS measurements. The black curve represents the intensity-weighted particle-size distribution derived from DLS.

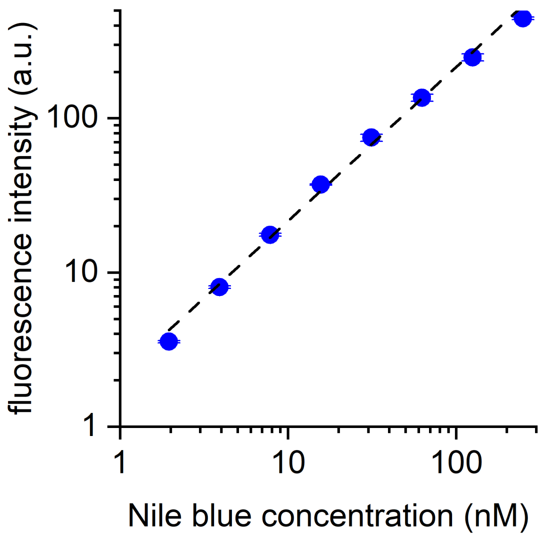

Figure S4: Linearity of fluorescence intensity measured by MDS. Dilution series of Nile blue in POPC nanodiscs encapsulated by Glyco-DIBMA (polymer/lipid mass ratio 2.0; Nile blue/lipid molar ratio 4.5×10^{-6}). The fluorescence emission intensity measured by MDS increases linearly with the concentration of the fluorescent dye over more than two orders of magnitude. The data shown correspond to lipid concentrations ranging from 0.44 mM to 56 mM. Note that the Nile blue labeling is particularly sparse in this case. Thus, even lower lipid concentrations can be used if the Nile blue/lipid ratio in the nanodiscs is increased.

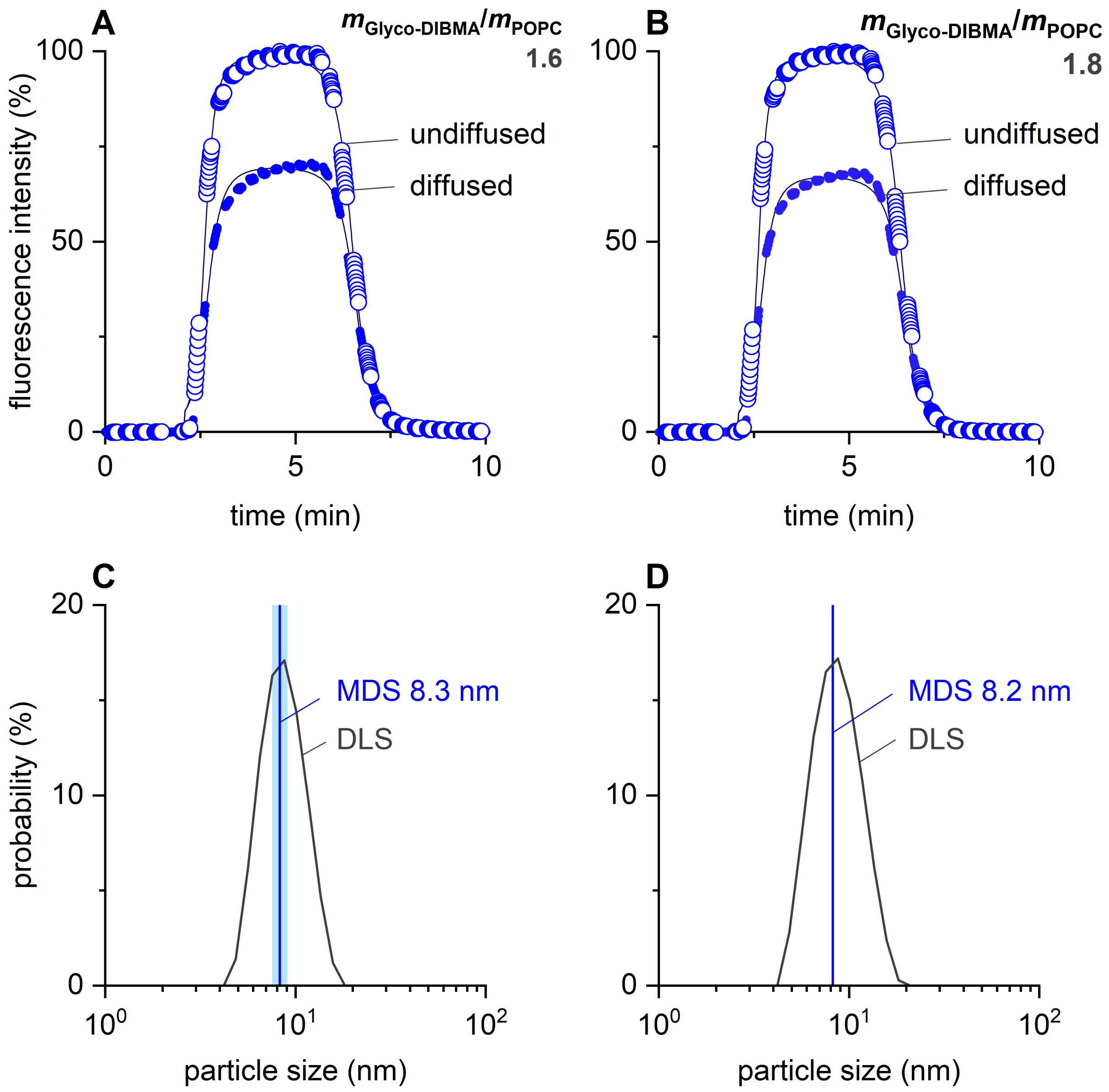

Figure S5: MDS and DLS of POPC nanodiscs solubilized with Glyco-DIBMA. (**A**,**B**) MDS traces of nanodiscs containing 1 mM POPC labeled with 25 nM Nile blue at two different polymer/lipid mass ratios. Shown are diffused (small, filled dots) and undiffused (large, unfilled dots) particles. The solid gray lines represent the corresponding fits. (**C**,**D**) Comparison of MDS and DLS measurements. The blue vertical lines indicate the sizes measured by MDS, while each of the light-blue shaded bands encompasses ±1 standard deviation determined from three MDS measurements. The black curves represent the intensity-weighted particle-size distributions derived from DLS.

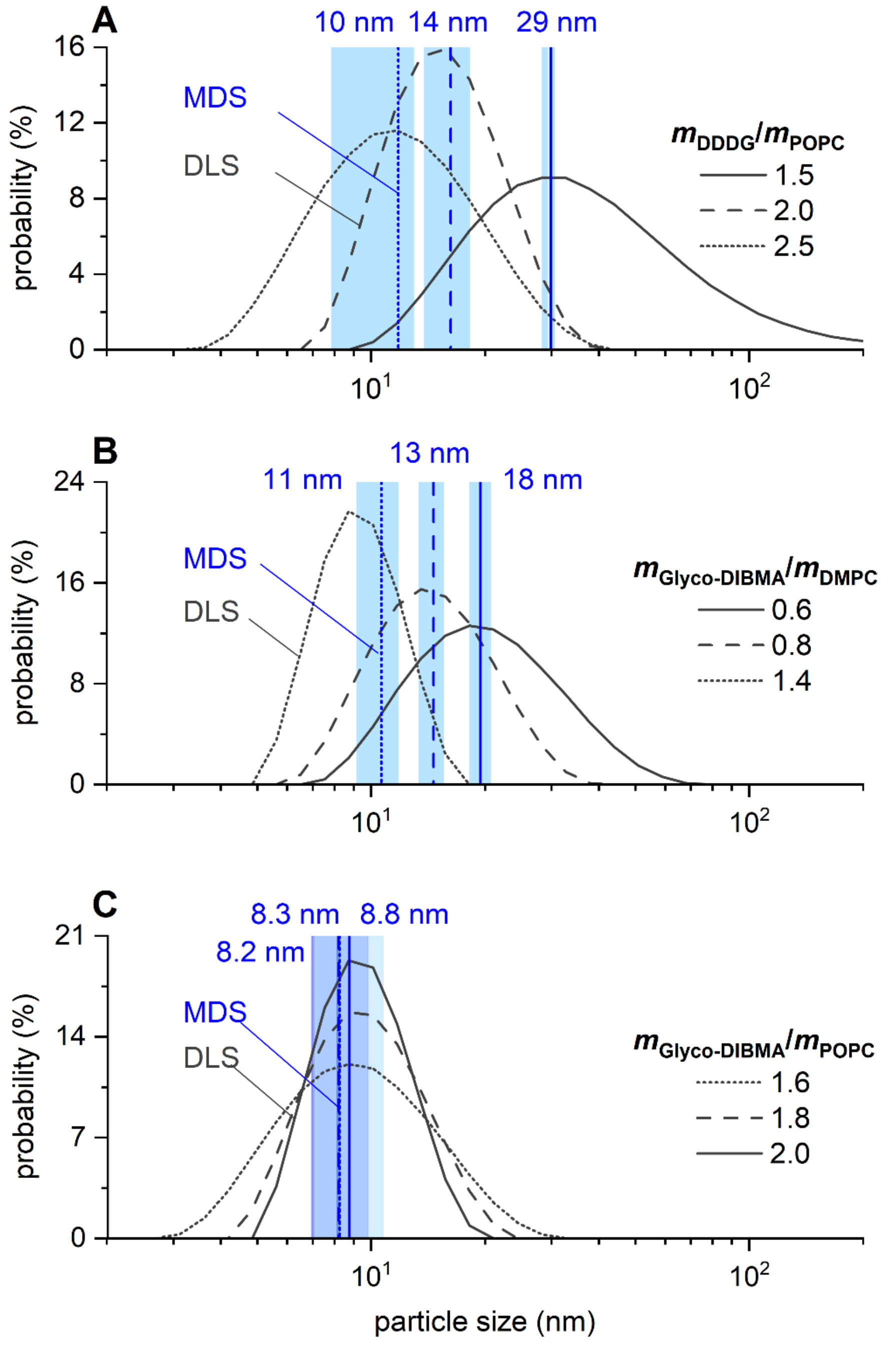

Figure S6: MDS and DLS of POPC and DMPC nanodiscs. In each case, three ratios of solubilizing agent to lipid were used to generate three different nanodisc sizes at a lipid concentration of 1 mM before labeling with 25–50 nM Nile blue. The blue vertical lines indicate the sizes measured by MDS, while each of the light-blue shaded bands encompasses ±1 standard deviation from three MDS measurements. The black curves represent the intensity-weighted particle-size distributions derived from DLS. (**A**) POPC nanodiscs solubilized with DDDG. (**B**) DMPC nanodiscs solubilized with Glyco-DIBMA. (**C**) POPC nanodiscs solubilized with Glyco-DIBMA.

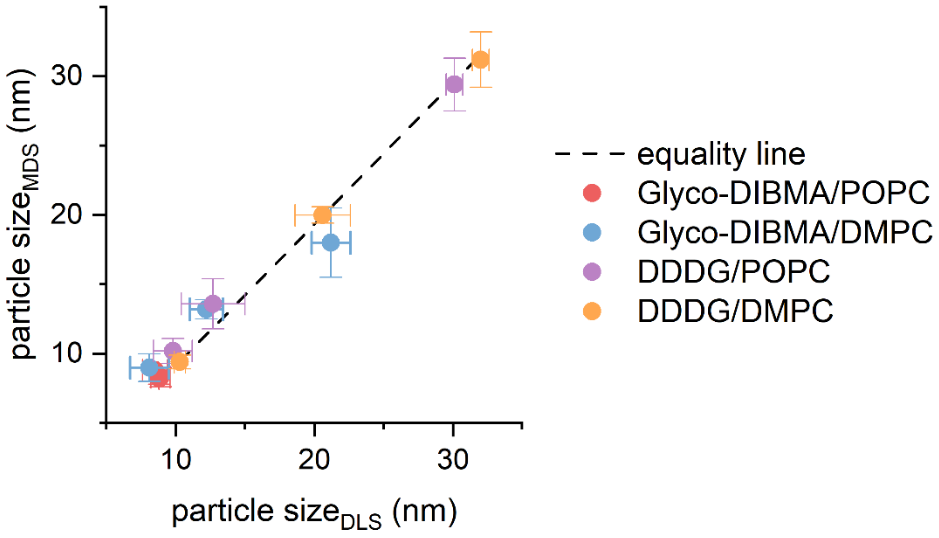

Figure S7: Correlation of particle sizes measured by MDS and DLS. Horizontal and vertical error bars indicate standard deviations (*n* = 3). Particle sizes obtained from the two methods are in good agreement.

Cells	Solubilizing agent	Particle size by MDS (nm)	Particle size by DLS (nm)
E. coli	Glyco-DIBMA	10.2 ± 0.1	115 ± 1
WM164	Glyco-DIBMA	11 ± 1	85 ± 41
WM164	DDDG	10 ± 3	113 ± 19

Table S1: MDS and DLS results for native nanodiscs made from cellular membranes. Given are mean sizes and standard deviations for nanodiscs made from *E. coli* (*n* = 3) or WM164 (*n* = 6) membranes.
